# Improvement in parameters of quality of life and uterine volume reduction after uterine fibroid embolization

**DOI:** 10.31744/einstein_journal/2020AO5458

**Published:** 2020-09-16

**Authors:** Nathalia Almeida Cardoso da Silva, Denis Szejnfeld, Rafael Kogan Klajner, Marcos Vinicius Maia da Mata, Ricardo Aun, Sergio Quilici Belczak

**Affiliations:** 1 Hospital Israelita Albert Einstein São Paulo SP Brazil Hospital Israelita Albert Einstein, São Paulo, SP, Brazil.; 2 Universidade Federal de São Paulo São Paulo SP Brazil Universidade Federal de São Paulo, São Paulo, SP, Brazil.; 3 Centro Universitário São Camilo São Paulo SP Brazil Centro Universitário São Camilo, São Paulo, SP, Brazil.

**Keywords:** Leiomyoma, Embolization, therapeutic, Quality of life, Treatment outcome

## Abstract

**Objective:**

To evaluate improvement in quality of life, reduction of uterine volume, and the correlation between these two variables after uterine fibroid embolization.

**Methods:**

Data on quality of life before and after uterine fibroid embolization were collected from 60 patients using the Uterine Fibroid Symptom – Quality of Life questionnaire. In 40 of these patients, uterine volume information on magnetic resonance imaging examinations performed before and after uterine fibroid embolization was collected, and compared using the nonparametric Wilcoxon test for paired data. Correlation between quality of life and uterine volume before and after procedure was measured using Spearman’s correlation coefficient.

**Results:**

There was significant improvement in quality of life after uterine fibroid embolization on Uterine Fibroid Symptom – Quality of Life questionnaire, in both subscales scores and the total score. There was a significant median reduction of -37.4% after uterine fibroid embolization, but no correlations between uterine volume and quality of life scores were found before or after embolization.

**Conclusion:**

Uterine embolization is an alternative to treat uterine fibroids, resulting in relief of symptoms and better quality of life. Although reduction in uterine volume plays an important role in the evaluation of therapeutic success, it does not necessarily have a definitive correlation with relief of symptoms.

## INTRODUCTION

Uterine fibroids, also called leiomyomas or fibroids, are the most prevalent tumors in gynecology. By definition, myoma is a benign neoplasm that affects 20% to 40% of women over the age of 30 years. Literature shows the prevalence of fibroids can reach up to 77%, with estimates of 6.5 tumors per affected uterus. Some factors such as age, parity, obesity, and ethnicity have been associated with a higher prevalence of fibroids. In addition, two or more affected first-degree relatives lead to an increase in its frequency of up to 2.2 times.^(1)^

Despite being mostly asymptomatic, it is estimated that 20% to 50% of fibroids do develop symptoms, which vary according to the size and location of the nodules. The number of fibroids can also interfere in intensity of symptoms. The presence of fibroids has a great impact on the quality of life of symptomatic patients. The main signs and symptoms that are usually caused by fibroids are menstrual changes, such as menorrhagia and/or metrorrhagia, resulting in anemia. In addition, there are also cases of intermenstrual bleeding, which tend to cause compressive symptoms, pain, and anatomical distortion of adjacent organs.^(2)^

The selection of the correct treatment, whether invasive or not, depends on the precise definition of fibroid size, number, and location. Magnetic resonance imaging (MRI) is a widely used test for uterine myomatosis because of its greater mapping ability when compared to ultrasonography, especially in cases of large (>375mL) or multiple (>4) fibroids.^(3)^Magnetic resonance imaging, as a non-operator-dependent exam, with low interobserver variability in routine interpretation of pre- and post-operative analyses, is a highly advantageous procedure.^(4)^

Regarding current treatment, there is a range of possible interventions, which may vary according to the patient’s wishes. Uterine embolization appears as a conservative choice for patients with symptomatic fibroids, whose surgical treatment is contraindicated, or who do not wish to undergo the surgical risks of a hysterectomy.^(5)^This method was first proposed as a treatment for myoma by Ravine, in France, in 1995. He found that in 90% of the cases, when choosing this treatment, patients had their myoma size reduced from 20% up to 70%.^(6-9)^

Great advantages in uterine fibroid embolization (UFE) have been highlighted in the literature: it is a minimally invasive procedure with positive results in preserving the uterus. Reduced size of fibroids and uterus, resumption of normal menstrual flow, shorter recovery time, and fertility preservation capability were reported.^(10,11)^ Studies reported the success rate for this method ranges from 87% to 90%, with a reduction in myoma size varying from 40% to 65%.^(7)^Other studies demonstrated the effectiveness of embolization in reducing symptoms and improving quality of life,^(8,9)^ including some results considered comparable to hysterectomy.^(12)^

There are few accounts of the Brazilian experience on this issue, but none evaluating quality of life using a specific and validated instrument.^(13)^In view of the poorer quality of life of symptomatic fibroid patients, and the increasing indications of therapeutic embolization, we evaluated the improvement in quality of life after the procedure. For a more technical assessment of the results of embolization, we measured the reduction in uterine volume on MRI and correlated with quality of life.

## OBJECTIVE

To assess improvement in quality of life and its correlation with uterine volume after uterine fibroid embolization.

## METHODS

A retrospective longitudinal study carried out with patients submitted to UFE for treatment of myoma at *Instituto Belczak de Cirurgia Vascular e Endovascular*, from January 2014 to December 2016. Patients were selected regardless of their age group. Patients with associated diseases causing bleeding and pelvic pain, such as myomatosis or endometriosis, or other associated abdominal conditions causing pain that could interfere in the results, and those submitted to uterine surgical procedures after embolization were excluded from the study.

Initially, 90 patients were contacted and invited to participate in the study. Those who showed interest in participating were called to the office of the principal investigator. Of these patients, 18 did not attend, and 7 informed they did not want to take part in the study. Of the remaining patients, two underwent myomectomy after embolization, two underwent hysterectomy, and one was diagnosed with uterine carcinomatosis and myoma, and were excluded from the study. Thus, 60 patients aged 35 to 53 years were finally included.

All patients underwent uterine embolization with microspheres. The technique was performed by right femoral artery puncture, followed by bilateral catheterization of the uterine artery with microcatheters, and release of 500μm to 700μm spheres. After spherical release, angiographic control was performed, and arteries not presenting distal flow were considered satisfactory.

### Quality of life assessment

During the first visit, all terms and objectives of the study were explained, and the Informed Consent Form was presented, explained in detail, and signed by the patients.

The Uterine Fibroid Symptom – Quality of Life (UFS-QoL) questionnaire^(14-16)^was used to collect retrospective data (prior to embolization) and current data (after procedure). The time between the procedure and the application of UFS-QoL ranged from 3 months to 3 years. In order to reduce recall bias, retrospective data from up to 3 years prior to the study were collected, taking into account that 50% of the critical details of a recognized event are unrecoverable after 5 years.^(17)^Some strategies, such as giving participants enough time before responding, to reflect and think through a sequence of events in their life history, were also adopted using standard protocols for data collection.^(18)^

The UFS-QoL contains 8 questions that quantify the severity of myomatous disease symptoms, and 29 questions related to quality of life in women’s health, focusing on concerns, energy, mood, control, self-awareness, and sexual function.

### Uterine volume assessment

Patients were also asked to bring their MRI exams performed before and after the surgery. In accordance to the service protocols, all patients underwent MRI before and 6 months after embolization for documentation purposes, and also because the decrease in uterine volume is used as reference to measure success of the procedure. Twenty patients who wanted to participate in the study, but did not bring their MRI or did not make their results available for analysis, were included exclusively in the analysis of symptoms and quality of life. Thus, 40 patients were assessed regarding uterine volume before and after UFE.

This study was conducted at *Instituto Belczak de Cirurgia Vascular e Endovascular*, according to CAAE: 70170817.3.0000.0071, and approval by the Research Ethics Committee of the *Hospital Israelita Albert Einstein* (HIAE), São Paulo (SP), Brazil, under number 2.180.478. All patients included in the study signed the Informed Consent Form.

### Statistical analysis

The comparisons between the pre- and post- timeframes for quality of life scores and uterine volume were made using the Wilcoxon non-parametric test for paired data. The correlation between post-embolization variation in the UFS-QoL questionnaire scores and uterine volume, as well as the correlation between greater previous uterine volume and worse quality of life in UFS-QoL before embolization, were analyzed using Spearman’s correlation test. Analyses were performed by means of the (SPSS) and R software, version 3.4.1, GAMLSS package, and a 5% level of significance was considered.

## RESULTS

### Quality of life

The scores for the symptom severity subscale, quality of life subscales, and total score of the UFS-QoL questionnaire applied to 60 patients were calculated according to the validated questionnaire for the Brazilian Portuguese. here was a significant improvement (p<0.001) between pre- and post-embolization in all scores of UFS-QoL, both for symptom severity and quality of life subscales (Table 1). There was a significant reduction in the symptom severity subscale scores as well as a significant increase in the other scores obtained after embolization.

**Table 1:** Total and subscale scores of the Uterine Fibroid Symptom *–* Quality of Life questionnaire before and after uterine fibroid embolization (n=60)

UFS-QoL	Evaluation		Variation	p value*
	Pre-embolization	Post-embolization		
Symptom severity				<0.001
Median (Q1-Q3)	68.8 (59.4-76.6)	9.4 (3.1-31.3)	-53.1 (-71.9- -29.7)	
(Min-Máx)	6.3-100.0	0.0-59.4	-81.3-3.1	
Concern				<0.001
Median (Q1-Q3)	25.0 (7.5-45.0)	92.5 (80.0-100.0)	60.0 (30.0-85.0)	
(Min-Máx)	0.0-100.0	25.0-100.0	-5.0-100.0	
Activities				<0.001
Median (Q1-Q3)	42.9 (25.0-57.1)	96.4 (85.7-100.0)	50.0 (32.1-67.9)	
(Min-Máx)	0.0-100.0	46.4-100.0	-14.3-89.3	
Energy/mood				<0.001
Median (Q1-Q3)	42.9 (26.8-57.1)	96.4 (83.9-100.0)	46.4 (30.4-67.9)	
(Min-Máx)	0.0-96.4	35.7-100.0	3.6-92.9	
Control				<0.001
Median (Q1-Q3)	40.0 (20.0-52.5)	90.0 (75.0-100.0)	50.0 (30.0-75.0)	
(Min-Máx)	0.0-100.0	5.0-100.0	5.0-90.0	
Self-awareness				<0.001
Median (Q1-Q3)	33.3 (16.7-50.0)	91.7 (75.0-100.0)	58.3 (25.0-66.7)	
(Min-Máx)	0.0-100.0	25.0-100.0	-8.3-100.0	
Sexual function				<0.001
Median (Q1-Q3)	37.5 (25.0-50.0)	100.0 (75.0-100.0)	50.0 (25.0-75.0)	
(Min-Máx)	0.0-100.0	50.0-100.0	-12.5-100.0	
Total score				<0.001
Median (Q1-Q3)	41.4 (22.0-50.0)	92.2 (83.2-97.8)	52.6 (30.2-73.3)	
(Min-Máx)	0.0-97.4	45.7-100.0	1.7-87.9	

* Wilcoxon’s test.

UFS-QoL: Uterine Fibroid Symptom – Quality of Life questionnaire; Q1: first quartile; Q3: third quartile.

### Uterine volume

Pre- and post-embolization MRI scans of 40 women submitted to the procedure were available. The three dimensions of the uterus were measured before and after embolization (Table 2), and the volume was calculated for each evaluation using the formula length × width × height × 0.523, assuming the ellipsoid shape of the uterus; differences between pre- and post-embolization measurements were calculated for all patients. There was a significant reduction by 37.4% (p<0.001) in the uterine volume after embolization (Table 2). One (2.5%) patient presented with an increase in uterine volume after UFE.

**Table 2:** Uterine volume (cm3) in pre- and post-embolization evaluations (n=40)

Evaluation	Median	Q1-Q3	Minimum-maximum
Pre-	343.4	217.8-564.0	79.9-145.0
Post-	173.5	107.5-314.4	60.6-296.0
Volume reduction	-141.3	-340.5- -59.3	-889.8-23.0
% reduction	-37.4	-51.9-27.9	-88.2-28.8

Wilcoxon’s test: p<0.001.

Q1: first quartile; Q3: third quartile.

### Correlation between uterine volume and Uterine Fibroid Symptom *–* Quality of Life questionnaire scores

To analyze the correlation between post-embolization variations, differences between pre- and post-embolization were considered as absolute values for both uterine volume and symptom severity scores. The correlation coefficients obtained indicated no correlation between changes in uterine volume and UFS-QoL scores. Also, no correlation was found between the uterine volume and UFS-QoL scores in the pre-embolization evaluation (Table 3).

**Table 3:** Correlation coefficients between uterine volume and Uterine Fibroid Symptom – Quality of Life questionnaire scores (n=40)

UFS-QoL items	Uterine volume and UFS-QoL scores	
	Post-embolization changes	Pre-embolization
Symptom severity	0.083 (0.612)	0.014 (0.933)
Concern	0.137 (0.399)	-0.147 (0.365)
Activity	-0.093 (0.566)	0.089 (0.583)
Energy/Mood	0.011 (0.947)	-0.097 (0.550)
Control	0.017 (0.918)	-0.159 (0.326)
Self-awareness	-0.007 (0.967)	-0.121 (0.458)
Sexual function	-0.205 (0.204)	0.013 (0.937)
Total score	0.029 (0.859)	-0.043 (0.792)

Results expressed as Spearman’s correlation coefficients (and p values).

UFS-QoL: Uterine Fibroid Symptom and Quality of Life questionnaire.

## DISCUSSION

Since embolization was proposed as an alternative treatment for uterine myomatosis in 1995, it has been possible to spare the reproductive organ through a less invasive procedure, improve symptoms and accelerate recovery. Although some important studies have already shown improved quality of life after UFE, this is the first Brazilian study using a validated instrument to assess uterine fibroid symptoms and quality of life, as the UFS-QoL questionnaire.

We found a significant median reduction of 53.1 points in the subscale of UFS-QoL symptom severity, and a significant improvement of 52.6 points in total quality of life score after UFE. These results were more expressive than those found in African women, after a 1-year follow-up, with reduction of 29.6 points in the symptom severity score, and an improvement of 35.7 points in quality of life, using the same questionnaire.^(19)^ Multicenter studies, such as FIBROID Registry, found reduction rates of 40.5 points in the severity of symptoms score, and a 39.67-point increase in quality of life.^(20)^Other large studies, including the EMMY (EMbolization versus hysterectoMY) trial, also reported improvement in quality of life at the 2-year follow-up after embolization, but used a less specific tool (the 36-Itens Short Form Health Survey questionnaire) for assessing quality of life.^(21)^ The high rates of improvement in symptoms and quality of life found in our study could be explained by the large proportion of women (81.6%) presenting with massive menstrual bleeding prior to embolization, a symptom considered as an isolated predictor for improvement of symptom scores.^(21,22)^

The improvement of sexual function after treatment of fibroids is a frequent concern for both women submitted to this procedure and their partners.^(23)^In this study, there was a significant improvement in the UFS-QoL sexual function subscale, in wich initial scores with a median of 37.5 points jumped to a median of 100 after UFE. It is important to emphasize that the UFS-QoL evaluates only some of the psychological aspects of sexual function, and does not collect data on desire, arousal, orgasm, and pain during sexual intercourse. Studies focused on these data reported improvement at the 1-year follow-up after embolization.^(23-25)^

It is worth mentioning that some strategies^(18)^ were used in this study for reducing recall bias, given that part of the data was collected up to 3 years after the UFE, which could result in more than 20% irretrievable critical details of a recognized event.^(17)^Although there is a higher risk of recall bias in retrospective studies, it is quite difficult to forget a significant clinical improvement after a surgical procedure in just 3 years, especially when impacting the quality of life so effectively.

There is controversy in literature about the influence of uterine size on the intensity of symptoms presented prior to embolization. Corroborating recent publications,^(9)^we did not find a significant correlation between these findings. The median uterine volume before embolization was 343.74cm^3^, similar to values observed in the EMMY trial (321cm^3^), but smaller when compared to the means reported in other large studies – approximately 500cm^3^ to 700cm^3^.^(9,19,21,23)^

There was a 37.1% reduction in uterine volume in our sample, slightly lower than values reported in other Brazilian and international studies.^(23,26-29)^Even with a smaller reduction in uterine volume, quality of life improvement scores were comparable to those of studies that had a greater reduction in uterine volume.^(30)^ As reported in previous studies,^(9,23)^there was no evidence of correlation between uterine volume changes and quality of life scores after UFE as well.

Therefore, significant reduction in uterine volume determines the technical success of UFE, without correlating with significant improvement in symptom severity/quality of life after the procedure. The efficacy of UFE as a less invasive alternative in the treatment of fibroids with improved quality of life seems to be proved. New studies should assess a possible better response to treatment, depending on the localization of the myoma (intramural, submucosal, or subserosal), or other factors that could predict treatment success.

Despite limitations in our study, such as the fact that it was not completely prospective, did not reach an ideal number of patients, and did not include some baseline information such as number, site, and size of myomas, and reasons for embolization (bleeding, pain, increased abdominal volume and/or infertility), our findings can contribute as a Brazilian experience to corroborate the effectiveness of UFE in improved quality of life, assessed through a validated questionnaire developed exclusively for such a condition.

## CONCLUSION

Uterine fibroid embolization is a therapy with significant reduction in uterine volume and improved quality of life, even though there is no correlation between uterine volume and symptom severity/quality of life before and after uterine fibroid embolization.
