# Supplementary material for: Improvement in parameters of quality of life and uterine volume reduction after uterine fibroid embolization
Source: Einstein (Sao Paulo). 2020 Sep 16;18:eAO5458. doi: 10.31744/einstein_journal/2020AO5458 (PMC9586426; doi:10.31744/einstein_journal/2020AO5458)
Supplement: Translation

# Translation: Melhora em parâmetros de qualidade de vida e redução do volume uterino após embolização de artérias uterinas

**Authors:** Nathalia Almeida Cardoso da Silva, Denis Szejnfeld, Rafael Kogan Klajner, Marcos Vinicius Maia da Mata, Ricardo Aun, Sergio Quilici Belczak

**Affiliations:** 1 Brasil Hospital Israelita Albert Einstein, São Paulo, SP, Brasil.; 2 Brasil Universidade Federal de São Paulo, São Paulo, SP, Brasil.; 3 Brasil Centro Universitário São Camilo, São Paulo, SP, Brasil.

**Keywords:** Leiomioma, Embolização terapêutica, Qualidade de vida, Resultado do tratamento

## Abstract

**Objetivo:**

Avaliar a melhora na qualidade de vida e a redução do volume uterino, além da correlação entre essas duas variáveis, após a embolização de artérias uterinas.

**Métodos:**

Foram coletados dados de 60 pacientes sobre qualidade de vida antes e depois da embolização de artérias uterinas com a aplicação do questionário *Uterine Fibroid Symptom* – *Quality of Life.* Informações sobre o volume uterino em exames de ressonância magnética realizada antes e depois do procedimento foram coletadas em 40 dessas pacientes e comparadas por meio de teste não paramétrico de Wilcoxon para dados pareados. A correlação entre qualidade de vida e volume uterino antes e depois do procedimento foi determinada pelo coeficiente de Spearman.

**Resultados:**

Houve melhora significativa na qualidade de vida das pacientes após embolização de artérias uterinas nos escores do questionário *Uterine Fibroid Symptom* – *Quality of Life*, tanto das subescalas como do total. Houve redução mediana significativa (-37,4%) no volume uterino após embolização de artérias uterinas, embora não tenha sido estabelecida qualquer correlação entre volume uterino e escores de qualidade de vida antes e depois da embolização.

**Conclusão:**

A embolização de artérias uterinas é alternativa para o tratamento de fibroide uterina, resultando na melhora dos sintomas e da qualidade de vida. Embora a redução do volume uterino seja fator importante na avaliação do sucesso terapêutico, não está necessariamente correlacionada com melhora de sintomas.

## INTRODUÇÃO

Miomas uterinos, também chamados de leiomiomas, são os tumores mais prevalentes na ginecologia. Por definição, leiomioma é uma neoplasia benigna que afeta 20% a 40% das mulheres com idade acima de 30 anos. A literatura mostra que a prevalência de miomas pode chegar a 77%, com estimativas de 6,5 tumores por útero afetado. Alguns fatores, como idade, paridade, obesidade e etnia, foram associados a uma maior prevalência de miomas. Além disso, dois ou mais parentes de primeiro grau afetados levam a aumento de até 2,2 vezes em sua frequência.^(1)^

Apesar de ser quase assintomático, estima-se que 20% a 50% de miomas apresentem sintomas, que podem variar segundo o tamanho e a localização dos nódulos. O número de miomas pode também interferir na intensidade de sintomas. A presença de leiomiomas tem grande impacto na qualidade de vida de pacientes sintomáticas. Os principais sinais e sintomas geralmente causados por miomas são alterações menstruais, como menorragia e/ou metrorragia, resultando em anemia. Ademais, existem casos de sangramento intermenstrual, que tende a causar sintomas compressivos, dor e distorção anatômica de órgãos adjacentes.^(2)^

A seleção do tratamento correto, invasivo ou não, depende da definição precisa do tamanho, do número e da localização do mioma. A ressonância magnética (RM) é um exame amplamente usado para miomatose uterina, por sua grande capacidade de mapeamento quando comparado com a ultrassonografia, em especial em casos de miomas grandes (>375mL) ou múltiplos (>4).^(3)^A RM como exame independente de operador, com baixa variabilidade interobservador na interpretação de rotina de análises pré- e pós-operatórias, é um procedimento altamente vantajoso.^(4)^

Quanto ao tratamento atual, há uma gama de possíveis intervenções, que variam segundo os desejos da paciente. A embolização de artérias uterinas aparece como opção conservadora para pacientes com miomas sintomáticos, cujo tratamento cirúrgico é contraindicado, ou que não desejam se submeter aos riscos cirúrgicos de uma histerectomia.^(5)^Este método foi proposto pela primeira vez como tratamento para mioma por Ravine, na França, em 1995. Ele encontrou que, em 90% dos casos, ao optar por esse tratamento, as pacientes tinham redução do tamanho de seus miomas de 20% até 70%.^(6-9)^

As principais vantagens na embolização de artérias uterinas (EAU) têm sido destacadas na literatura: é um procedimento minimamente invasivo com resultados positivos na preservação do útero. O tamanho reduzido de miomas e de útero, a retomada do fluxo menstrual normal, o tempo de recuperação mais curto e a capacidade de preservar a fertilidade foram descritos.^(10,11)^ Estudos relatam que a taxa de sucesso para esse método varia de 87% a 90%, com redução no tamanho do mioma de 40% a 65%.^(7)^Outros estudos demonstraram a efetividade da embolização na redução de sintomas e a melhora da qualidade de vida,^(8,9)^ incluindo resultados considerados comparáveis aos da histerectomia.^(12)^

Há poucos relatos da experiência brasileira sobre este tópico, e nenhum avalia a qualidade de vida por meio de um instrumento específico e validado.^(13)^ Considerando a pior qualidade de vida das pacientes com miomas sintomáticos e o aumento de indicações para embolização terapêutica, avaliamos a melhora na qualidade de vida após o procedimento. Para avaliação mais técnica dos resultados de embolização, medimos a redução do volume uterino por meio de RM e correlacionamos com qualidade de vida.

## OBJETIVO

Avaliar a melhora na qualidade de vida e em sua correlação com volume uterino após a embolização de artérias uterinas.

## MÉTODOS

Estudo retrospectivo longitudinal realizado com pacientes submetidas à EAU para o tratamento de leiomioma no Instituto Belczak de Cirurgia Vascular e Endovascular, de janeiro de 2014 a dezembro de 2016. As pacientes foram selecionadas independentemente da faixa etária. Pacientes com doenças associadas que causam sangramento e dor pélvica, como miomatose ou endometriose, ou outras doenças abdominais que causam dor que pudesse interferir nos resultados, e aquelas submetidas a procedimentos cirúrgicos uterinos após a embolização foram excluídas do estudo.

Inicialmente, 90 pacientes foram contatadas e convidadas a participar no estudo. Aquelas que demonstraram interesse em participar foram chamadas ao consultório do investigador principal. Destas pacientes, 18 não compareceram, e 7 informaram que não desejavam participar do estudo. Das pacientes restantes, duas foram submetidas à miomectomia após embolização, duas passaram por histerectomia e uma foi diagnosticada com carcinomatose uterina além do mioma, tendo sido excluídas do estudo. Assim, 60 pacientes, entre 35 e 53 anos, foram finalmente incluídas.

Todas as pacientes se submeteram à embolização uterina com microesferas. A técnica foi feita por meio de punção da artéria femoral direita, seguida de cateterismo bilateral da artéria uterina com microcateteres e liberação de esferas de 500μm a 700μm. Após a liberação das microesferas, realizou-se controle angiográfico, e as artérias que não apresentaram fluxo distal foram consideradas satisfatórias.

### Avaliação de qualidade de vida

Durante a primeira visita, todos os termos e objetivos do estudo foram esclarecidos, e o Termo de Consentimento Livre e Esclarecido foi apresentado, explicado em detalhes, e assinado pelas pacientes.

O questionário *Uterine Fibroid Symptom – Quality of Life* (UFS-QoL)^(14-16)^foi aplicado para coletar dados retrospectivos (antes da embolização) e dados atuais (após o procedimento). O tempo entre o procedimento e a aplicação do UFS-QoL variou de 3 meses a 3 anos. Para poder reduzir o viés de *recall*, dados retrospectivos de até 3 anos antes do estudo foram coletados, levando em consideração que 50% dos detalhes críticos de um evento reconhecido são irrecuperáveis após 5 anos.^(17)^ Algumas estratégias, como conceder às participantes tempo suficiente antes de responder, para refletir e pensar na sequência de acontecimentos em sua história de vida, também foram adotadas, usando protocolos padrão para coleta de dados.^(18)^

O UFS-QoL contém 8 questões que quantificam a gravidade dos sintomas da doença miomatosa e 29 questões relacionadas à qualidade de vida na saúde feminina, enfocando preocupações, energia, humor, controle, autoconsciência e função sexual.

### Avaliação do volume uterino

As pacientes também foram solicitadas a trazer os resultados de seus exames de RM feitos antes e depois da cirurgia. De acordo com os protocolos do serviço, todas as pacientes foram submetidas à RM antes e 6 meses depois da embolização para fins de documentação, e também porque a diminuição do volume urinário é usada como referência para medir o sucesso do procedimento. Foram incluídas exclusivamente na análise de sintomas e qualidade de vida 20 pacientes que desejavam participar do estudo, mas não trouxeram os resultados de RM ou não disponibilizaram seus resultados para análises. Desse modo, 40 pacientes foram avaliadas em relação ao volume urinário antes e depois da EAU.

Este estudo foi conduzido no Instituto Belczak de Cirurgia Vascular e Endovascular, protocolo CAAE: 70170817.3.0000.0071, e aprovado pelo Comitê de Ética em Pesquisa do Hospital Israelita Albert Einstein (HIAE), São Paulo (SP), sob número 2.180.478. Todos as pacientes incluídas no estudo assinaram o Termo de Consentimento Livre e Esclarecido.

### Análise estatística

As comparações entre os momentos pré- e pós- para os escores de qualidade de vida e volume uterino foram feitos usando o teste de Wilcoxon não paramétrico para dados pareados. As correlações entre a variação nos escores pós-embolização e volume uterino do UFS-QoL, e entre o volume uterino prévio e pior qualidade de vida no questionário antes da embolização foram analisadas usando o teste de correlação de Spearman. As análises foram feitas com os programas (SPSS) e R, versão 3.4.1, pacote GAMLSS, com nível de significância de 5%.

## RESULTADOS

### Qualidade de vida

Os escores para a subescala de gravidade do sintoma, as subescalas de qualidade de vida e o escore total do UFS-QoL aplicados a 60 pacientes foram calculados segundo o questionário validado para português do Brasil. Houve melhora significante (p<0,001) entre a pré- e pós-embolização em todos os escores do UFS-QoL, tanto para a gravidade do sintoma e como para as subescalas de qualidade de vida (Tabela 1). Houve significante redução nos escores de gravidade dos sintomas, além de melhora importante em outros escores após a embolização.

**Tabela 1:** Escores total e das subescalas do questionário *Uterine Fibroid Symptom – Quality of Life* antes e depois da embolização de artérias uterinas (n=60)

UFS-QoL	Avaliação		Variação	Valor de p*
	Pré-embolização	Pós-embolização		
Gravidade dos sintomas				<0,001
Mediana (Q1-Q3)	68,8 (59,4-76,6)	9,4 (3,1-31,3)	-53,1 (-71,9- -29,7)	
(Min-Máx)	6,3-100,0	0,0-59,4	-81,3-3,1	
Preocupação				<0,001
Mediana (Q1-Q3)	25,0 (7,5-45,0)	92,5 (80,0-100,0)	60,0 (30,0-85,0)	
(Min-Máx)	0,0-100,0	25,0-100,0	-5,0-100,0	
Atividades				<0,001
Mediana (Q1-Q3)	42,9 (25,0-57,1)	96,4 (85,7-100,0)	50,0 (32,1-67,9)	
(Min-Máx)	0,0-100,0	46,4-100,0	-14,3-89,3	
Energia/humor				<0,001
Mediana (Q1-Q3)	42,9 (26,8-57,1)	96,4 (83,9-100,0)	46,4 (30,4-67,9)	
(Min-Máx)	0,0-96,4	35,7-100,0	3,6-92,9	
Controle				<0,001
Mediana (Q1-Q3)	40,0 (20,0-52,5)	90,0 (75,0-100,0)	50,0 (30,0-75,0)	
(Min-Máx)	0,0-100,0	5,0-100,0	5,0-90,0	
Autoconsciência				<0,001
Mediana (Q1-Q3)	33,3 (16,7-50,0)	91,7 (75,0-100,0)	58,3 (25,0-66,7)	
(Min-Máx)	0,0-100,0	25,0-100,0	-8,3-100,0	
Função sexual				<0,001
Mediana (Q1-Q3)	37,5 (25,0-50,0)	100,0 (75,0-100,0)	50,0 (25,0-75,0)	
(Min-Máx)	0,0-100,0	50,0-100,0	-12,5-100,0	
Escore total				<0,001
Mediana (Q1-Q3)	41,4 (22,0-50,0)	92,2 (83,2-97,8)	52,6 (30,2-73,3)	
(Min-Máx)	0,0-97,4	45,7-100,0	1,7-87,9	

* Teste de Wilcoxon.

UFS-QoL: *Uterine Fibroid Symptom – Quality of Life*; Q1: primeiro quartil; Q3: terceiro quartil.

### Volume uterino

Estavam disponíveis os resultados de RM pré- e pós-embolização de 40 mulheres submetidas ao procedimento. As três dimensões do útero foram medidas antes e depois da embolização (Tabela 2), e o volume foi calculado para cada avaliação usando a fórmula comprimento × largura × altura × 0,523, considerando a forma elipsoide do útero; as diferenças entre as medidas pré- e pós-embolização foram calculadas para todas as pacientes. Houve redução significante de 37,4% (p<0,001) no volume uterino após a embolização (Tabela 2). Uma (2,5%) paciente apresentou aumento do volume uterino após EAU.

**Tabela 2:** Volume uterino (cm3) em avaliações pré- e pós-embolização (n=40)

Avaliação	Mediana	Q1-Q3	Mínima-máxima
Pré	343,4	217,8-564,0	79,9-145,0
Pós	173,5	107,5-314,4	60,6-296,0
Redução de volume	-141,3	-340,5-59,3	-889,8-23,0
Redução, %	-37,4	-51,9-27,9	-88,2-28,8

Teste de Wilcoxon: p<0,001.

Q1: primeiro quartil; Q3: terceiro quartil.

### Correlação entre volume uterino e escores do *Uterine Fibroid Symptom* – *Quality of Life*

Para analisar a correlação entre as variações pós-embolização, diferenças entre pré e pós-embolização foram consideradas como valores absolutos para volume uterino e escores de gravidade do sintoma. Os coeficientes de correlação obtidos não indicaram correlação entre alterações no volume uterino e escores do UFS-QoL. Além disso, nenhuma correlação foi encontrada entre o volume uterino e os escores na avaliação pré-embolização (Tabela 3).

**Tabela 3:** Coeficientes de correlação entre volume uterino e escores do questionário *Uterine Fibroid Symptom – Quality of Life* (n=40)

Itens do UFS-QoL	Volume uterino e escores de UFS-QoL	
	Alterações pós-embolização	Pré-embolização uterina
Gravidade do sintoma	0,083 (0,612)	0,014 (0,933)
Preocupação	0,137 (0,399)	-0,147 (0,365)
Atividade	-0,093 (0,566)	0,089 (0,583)
Energia/humor	0,011 (0,947)	-0,097 (0,550)
Controle	0,017 (0,918)	-0,159 (0,326)
Autoconsciência	-0,007 (0,967)	-0,121 (0,458)
Função sexual	-0,205 (0,204)	0,013 (0,937)
Escore total	0,029 (0,859)	-0,043 (0,792)

Resultados expressos como coeficientes de correlação de Spearman (valor de p).

UFS-QoL: Questionário *Uterine Fibroid Symptom – Quality of Life*.

## DISCUSSÃO

Desde que a embolização foi proposta como tratamento alternativo para miomatose uterina em 1995, é possível preservar o órgão reprodutivo por meio de um procedimento menos invasivo, melhorar sintomas e acelera a recuperação. Embora alguns importantes estudos já tenham demonstrado melhor qualidade de vida após EAU, este é o primeiro estudo brasileiro que usa um instrumento validado para analisar os sintomas de miomas uterinos e qualidade de vida como questionário UFS-QoL.

Encontramos significante redução mediana de 53,1 pontos na subescala de gravidade dos sintomas do UFS-QoL, além de significante melhora de 52,6 pontos no escore total de qualidade de vida após EAU. Esses resultados foram mais expressivos do que aqueles encontrados em mulheres africanas, após acompanhamento de 1 ano, com redução de 29,6 pontos no escore de gravidade de sintoma e melhora de 35,7 pontos em qualidade de vida, usando o mesmo questionário.^(19)^ Estudos multicêntricos, como o FIBROID Registry, encontraram taxas de 40,5 pontos no escore de gravidade dos sintomas e aumento de 39,67-pontos na qualidade de vida.^(20)^ Outros grandes estudos, incluindo o ensaio EMMY (*EMbolization versus hysterectoMY*), também descreveram melhora na qualidade de vida no acompanhamento de 2 anos após a embolização, mas utilizaram ferramenta menos específica (o questionário *36-Itens Short Form Health Survey*) para avaliar a qualidade de vida.^(21)^

As altas taxas de melhora em sintomas e qualidade de vida encontradas em nosso estudo podem ser explicadas pela grande proporção de mulheres (81,6%) que apresentava sangramento menstrual maciço antes da embolização, um sintoma considerado preditor isolado de otimização dos escores de sintoma.^(21,22)^

A melhora de função sexual após o tratamento de miomas é preocupação frequente para as mulheres submetidas a este procedimento e seus parceiros.^(23)^Neste estudo, houve melhora significante na subescala de função sexual do UFS-QoL, na qual os escores iniciais com mediana de 37,5 pontos aumentaram para mediana de 100 após a EAU. É importante enfatizar que o UFS-QoL avalia apenas alguns dos aspectos psicológicos da função sexual, e não coleta dados sobre desejo, excitação, orgasmo e dor durante a relação sexual. Estudos focados nesses dados relataram melhora na avaliação de 1 ano após a embolização.^(23-25)^

Ainda, algumas estratégias^(18)^ foram usadas neste estudo para reduzir o viés de *recall*, considerando que parte dos dados foi coletada até 3 anos após a EAU, o que poderia resultar em mais de 20% de detalhes críticos irrecuperáveis de um evento reconhecido.^(17)^ Embora exista um risco maior de viés de *recall* em estudos retrospectivos, é muito difícil se esquecer de uma melhora clínica significante após um procedimento cirúrgico em apenas 3 anos, especialmente quando o um impacto é tão efetivo na qualidade de vida.

Existe controvérsia na literatura sobre a influência do tamanho uterino na intensidade dos sintomas apresentados antes da embolização. Corroborando publicações mais recentes,^(9)^não encontramos correlação significante entre esses achados. A mediana do volume uterino antes da embolização foi de 343,74cm^3^, similar aos valores observados no ensaio EMMY (321cm^3^), mas menor quando comparado às médias relatadas em outros grandes estudos – cerca de 500cm^3^ a 700cm^3^.^(9,19,21,23)^

Houve redução de 37,1% no volume uterino na nossa amostra, discretamente menor que os valores relatados em outros estudos brasileiros e internacionais.^(23,26-29)^Mesmo com menor redução em volumes uterino, os escores de melhora de qualidade de vida foram comparáveis aos de estudos que tiveram maior redução em volume uterino.^(30)^ Como relatado em estudos prévios,^(9,23)^não houve evidência de correlação entre alterações de volume uterino e escores de qualidade de vida após EAU.

Assim, uma significante redução no volume uterino determina o sucesso técnico de EAU, sem correlacionar com melhora significante na gravidade do sintoma/qualidade de vida após o procedimento. A eficácia da EAU como alternativa menos invasiva no tratamento de miomas com qualidade de vida melhorada parece ter sido comprovada. Novos estudos devem avaliar uma possível melhor resposta ao tratamento, dependendo da localização do mioma (intramural, submucosa ou subserosa) ou de outros fatores que poderiam predizer o sucesso do tratamento.

Nosso estudo apresentou limitações, como o fato de não ser completamente prospectivo, não atingir o número ideal de pacientes e não incluir algumas informações iniciais, como número, local e tamanho dos miomas, além dos motivos para embolização (sangramento, dor, volume abdominal aumentado e/ou infertilidade). Mesmo assim, nossos achados podem contribuir como experiência brasileira para corroborar a efetividade da EAU na melhoria de qualidade de vida, avaliada por meio de um questionário validado desenvolvido exclusivamente para esta doença.

## CONCLUSÃO

A embolização de artérias uterinas é um tratamento com importante redução no volume uterino e melhoria na qualidade de vida, mesmo que não haja correlação entre volume uterino e gravidade de sintoma/qualidade de vida antes e depois da embolização das artérias uterinas.
